# Neuromuscular blockade management in the critically Ill patient

**DOI:** 10.1186/s40560-020-00455-2

**Published:** 2020-05-24

**Authors:** J. Ross Renew, Robert Ratzlaff, Vivian Hernandez-Torres, Sorin J. Brull, Richard C. Prielipp

**Affiliations:** 1 Department of Anesthesiology and Perioperative Medicine, Mayo Clinic Florida, 4500 San Pablo Road, Jacksonville, FL 32224 USA; 2 Department of Critical Care Medicine, Mayo Clinic, Jacksonville, FL USA; 3 Department of Anesthesiology, University of Minnesota Medical School, Minneapolis, MN USA

**Keywords:** Intensive care unit, Critical care, Neuromuscular blocking agents, Neuromuscular blockade, Neuromuscular monitoring, Pharmacologic antagonism

## Abstract

Neuromuscular blocking agents (NMBAs) can be an effective modality to address challenges that arise daily in the intensive care unit (ICU). These medications are often used to optimize mechanical ventilation, facilitate endotracheal intubation, stop overt shivering during therapeutic hypothermia following cardiac arrest, and may have a role in the management of life-threatening conditions such as elevated intracranial pressure and status asthmaticus (when deep sedation fails or is not tolerated). However, current NMBA use has decreased during the last decade due to concerns of potential adverse effects such as venous thrombosis, patient awareness during paralysis, development of critical illness myopathy, autonomic interactions, and even residual paralysis following cessation of NMBA use.

It is therefore essential for clinicians to be familiar with evidence-based practices regarding appropriate NMBA use in order to select appropriate indications for their use and avoid complications. We believe that selecting the right NMBA, administering concomitant sedation and analgesic therapy, and using appropriate monitoring techniques mitigate these risks for critically ill patients. Therefore, we review the indications of NMBA use in the critical care setting and discuss the most appropriate use of NMBAs in the intensive care setting based on their structure, mechanism of action, side effects, and recognized clinical indications. Lastly, we highlight the available pharmacologic antagonists, strategies for sedation, newer neuromuscular monitoring techniques, and potential complications related to the use of NMBAs in the ICU setting.

## Introduction

The introduction of neuromuscular blocking agents to the ICU provides intensivists a unique capability in the management of critically ill patients. As with any therapy, however, the use of NMBAs has inherent risks, particularly when providers are unfamiliar with the nuances of selecting the appropriate agent, monitoring the depth of neuromuscular blockade, and ensuring adequate skeletal muscle recovery once NMBA therapy has ceased. Optimal neuromuscular blockade management has challenged clinicians for decades, despite the frequent use of NMBAs in clinical practice [1]. Complications associated with the NMBA use can be particularly concerning in the critical care setting, as intensivists typically administer NMBAs to critically ill patients with multi-organ system derangements for long periods of time resulting in greater accumulation of NMB drug and drug metabolites. The impact of such “off-label” use of NMBAs in the ICU is still being investigated. The Society of Critical Care Medicine (SCCM) developed guidelines addressing optimal practice based on the available evidence to address these concerns [2–4].

While guidelines can help clinicians navigate many clinical scenarios, these recommendations are often limited by the lack of well-designed prospective trials. Ultimately, a thorough understanding of neuromuscular blockade management can equip clinicians to deal with scenarios that fall outside of the scope of medical specialty guidelines. This review provides up-to-date evidence to aid clinicians in selecting the right scenarios for establishing neuromuscular blockade in the ICU as well as choosing the optimal agent for such scenarios. Additionally, we will review methods to determine the level of neuromuscular blockade, the use of NMBA antagonists, and the optimal methods to confirm an adequate neuromuscular recovery and avoid prolonged residual weakness in this vulnerable patient population.

## Indications

In 2016, a task force comprising 17 members from the Society of Critical Care Medicine (SCCM) proposed updated and comprehensive recommendations for the use of neuromuscular blocking agents in the critically ill patient (Table 1) [4]. The authors expanded upon previous recommendations from 2002 [2] while utilizing the Grading of Recommendations Assessment, Development, and Evaluation (GRADE) system [5] to comment on the quality-of-evidence for each recommendation. These recommendations can be utilized in a variety of critical care settings that require neuromuscular blockade; however, these guidelines are limited by the relative paucity of definitive literature investigating neuromuscular blockade in the unique critically ill patient population.

**Table 1:** Clinical practice guidelines for the sustained neuromuscular blockade in the adult critically ill patient [3]

Clinical practice(s)	Strength of Recommendation
• Scheduled eye care with lubrication and eyelid closure	Strong recommendation
• Continuous infusion of NMBA rather than intermittent boluses • Avoid use in status asthmaticus • Trial of NMBA in life-threatening situations with hypoxemia, respiratory acidosis, and hemodynamic compromise • May be used to manage overt shivering in therapeutic hypothermia • PNS with inclusive clinical assessment may be a useful tool for determining the depth of blockade • PNS should not be used alone (without clinical assessments) in patients receiving a continuous infusion of NMBAs • Implementation of a structured physiotherapy regimen • Target blood glucose level < 180 mg/dL • Dose NMBA based on ideal body weight or adjusted boy weight (rather than actual)	Weak recommendation
• PNS can be used with clinical assessment in patients undergoing therapeutic hypothermia • Protocols should be utilized to guide NMBA administration in patients undergoing therapeutic hypothermia • Analgesic and sedative drugs should be used before and during neuromuscular blockade • Implement measures to reduce risk of unintended extubation in patients receiving NMBAs • Reduce dosing in patients with myasthenia gravis based on PNS use • Discontinue NMBAs prior to determining brain death	Good practice based on expert opinion with insufficient evidence

*NMBA* neuromuscular blocking agents, *PNS* peripheral nerve stimulator

### Facilitation of tracheal intubation

Endotracheal intubation in the ICU is a more challenging endeavor than in the controlled environment of the operating room (OR), and the risk of a “failed intubation” is several-fold greater in the ICU [6]. Unlike the OR where the primary objective of tracheal intubation is to secure the airway after induction of anesthesia, the procedural objective in the ICU is to secure the airway as a life-saving intervention in a patient with current or impending respiratory failure [7]. Endotracheal intubation in the critical care setting is associated with significant complications such as severe hypotension, hypoxemia, and even cardiac arrest [7–9]. Such complications can occur up to 25% of the time [10]. Moreover, when managing the difficult airway, the intensivist rarely has the option to awaken the patient during the scenario of “failed intubation” as suggested by the American Society of Anesthesiologists’ (ASA) difficult airway algorithm [11].

Nonetheless, the use of NMBAs is an important adjunct to facilitate tracheal intubation as these drugs can create better conditions during laryngoscopy [12]. In addition, the NMBA use can significantly decrease airway trauma associated with this procedure and facilitate securing the airway in fewer attempts [13]. Succinylcholine and rocuronium are the two agents typically utilized when the neuromuscular blockade is desired to rapidly facilitate tracheal intubation. While succinylcholine provides rapid and reliable neuromuscular blockade, higher doses of rocuronium (1.2 mg/kg or 4× the effective dose that decreases the twitch by 95% from baseline [ED_95_]) can have a similar mean onset time (although a slightly wider range of onset times), a characteristic that makes this agent suitable for rapid sequence induction and intubation (RSII) [14]. Higher doses of rocuronium result in a much longer duration of action than succinylcholine, increasing concerns about its use in the patient with a difficult airway. However, high-dose rocuronium can be antagonized with sugammadex (at a dose of 16 mg/kg) after 3 min in the “can’t intubate/can’t ventilate” scenario [15]. This pharmacologic reversal, however, does not ensure the avoidance of dangerous periods of hypoxia (or hypoventilation due to opioid or sedative drugs co-administered), and rapid, appropriate airway management targeted at establishing airway patency remains paramount [16].

## Airway management of the ICU patient

Management of the airway of ICU patients presents multiple and varied challenges, as it is one of the most commonly performed procedures in this setting. The identification of the difficult airway is paramount, and its incidence may be over 11% [17]. Serious adverse events from attempted tracheal intubation performed in the ICU patients occur in up to 40% of cases [18]. In order to identify patients at risk of difficult intubation, some investigators have recommended development of simple scores that can be applied at bedside. One such scale, the MACOCHA Score, consists of a total of 12 points (see Table 2), and combines patient, patient pathology, and operator factors to differentiate between difficult and nondifficult intubation patients in the ICU [17]. Patient factors included are Mallampati score of III or IV, the presence of obstructive sleep apnea, reduced mobility of the cervical spine, and limited mouth opening. Patient pathology factors were severe hypoxia and coma, while the operator factor was the presence of a nonanesthesiologist for airway management. The scale for identification of risk factors for difficult airway/intubation in critically ill patients by nonanesthesiologist trainees was further refined and validated in a prospective, observational single-center study [19].

**Table 2:** Score calculation worksheet, MACOCHA Scale

	Points
**(M) Mallampati > 2**	**5**
**(A) Obstructive sleep apnea**	**2**
**(C) Cervical spine limitation**	**1**
**(O) Limited mouth opening**	**1**
**(C) Coma**	**1**
**(H) Severe hypoxemia**	**1**
**(A) Non-anesthesiologist performing intubation**	**1**
**Total**	**12**

Adapted from De Jong et al. Am J Respir Crit Care Med 2013 [17]

Despite the availability of indicators of difficult airway in ICU patients, however, a recent French survey found that 43% of intubating operators were still not fully proficient in the technique, with 18.8% of them having had no intubation training, or only basic training, such as lectures or observation [18]. This survey also reported that although video laryngoscopy is available in most of the French ICUs, its use was reserved for management of the difficult airway patients [18]. Remarkably, the vast majority (83%) of intensivists had placed less than a total of 10 laryngeal mask airways, and half had performed less than 10 intubations using fiberoptic bronchoscopy, despite the fact that a majority (87%) of clinicians expressed a desire to participate in high fidelity mannequin simulations [20]. A Spanish national survey reported that of the 101 ICUs that responded, three quarters had no tracheal intubation or no difficult airway protocols [21]. The authors thus called for the implementation of changes in the ICU that include prospective identification of experts in management of the difficult airway and the development of specific guidelines for management of the ICU patient with difficult airway [21]. In Japan, difficult airway management carts are largely unavailable in the ICU, and capnography to confirm correct tracheal tube placement is used in only slightly over half of the patients [22]. In the UK, 6.3% of ICU patients were judged to have an increased risk of airway complications, but only 19% of them had a plan in place for management of the difficult airway [23]. In Australia and New Zealand, only a small minority of ICUs identify patients with “critical airways,” and only 8% have specific protocols for care of these high-risk patients [24].

The ICU patient with a difficult airway poses a significant challenge not only when the airway needs to be secured; the same precautions and potential for adverse events remain at the time of tracheal extubation. The Royal College of Anaesthetists’ 4th National Audit Project (NAP 4) has reinforced the importance of optimal airway management in the ICU environment, has underscored the need for appropriate guidelines and strategies for the safe extubation of the trachea in patients with a potentially difficult airway, and has proposed key anesthetic principles for safe airway management (Table 3) [25].

**Table 3:** Key anesthetic principles for airway management strategies in ICU patients

1. Oxygenation, not intubation, is the priority at all times including during tracheal extubation.	
2. Airway equipment should be purchased with the least experienced potential user in mind, and not the most experienced (i.e., ideally, devices should be intuitive and user-friendly, requiring a short training period).	
3. Devices should have sufficient evidence from reliable research to support their clinical role.	
4. Rescue devices should have a close to 100% success rate to ensure the minimal number of steps when securing the airway. A device with a high success rate in routine use may have a lower success rate when used as a rescue maneuver, especially when the difficult airway is unexpected. Urgency and operator’s anxiety of impending patient morbidity or mortality is likely to hinder the success of any device.	
5. Devices should be trialed over an adequate period of time (several weeks or months in most cases, and a sufficient number of times, preferably more than 50) to ensure that they are used for a variety of airway problems and by an adequate cross-section of staff.	
6. To be successful, extubation should be planned in a similar manner to intubation. To be more specific, extubation techniques should be tailored to the type of expected airway difficulties. Preparation for re-intubation should be part of the extubation management plan with a clear indication of when an intervention is or is not working and when to seek alternative methods.	
7. Technical and non-technical training in all clinical environments must follow the implementation of new airway management and oxygenation devices.	

### Facilitation of mechanical ventilation

In the ICU, NMBAs are also commonly used for the facilitation of mechanical ventilation. The current SCCM clinical practice guidelines [4] suggest that an NMBA be administered by continuous intravenous infusion early in the course of acute lung injury for patients with a partial pressure of oxygen to fraction of inspired oxygen (PaO_2_/FiO_2_) ratio less than 150 (*weak recommendation with moderate quality of evidence*). Indeed, patients with acute respiratory distress syndrome (ARDS) are unlikely to oxygenate or ventilate optimally with sedation/analgesia regimens alone. Gainnier et al. conducted a multi-center, prospective controlled randomized trial and found that the use of NMBAs during a 48-h period in ARDS patients was associated with a sustained improvement in oxygenation [26]. In the ACURASYS trial, Pappazian et al. found that in patients with severe ARDS, early administration of cisatracurium continuously for 48 h improved the adjusted 90-day survival, decreased the risk of barotrauma, and increased the time off the ventilator without increasing muscle weakness [27]. However, more recent results from the Reevaluation of Systemic Early Neuromuscular Blockade (ROSE) trial failed to show reductions in mortality when NMBAs were administered in moderate-severe ARDS [28]. While cisatracurium has been shown to possess anti-inflammatory properties in animal models [29], its clinically relevant benefit likely involves avoidance of ventilator dyssynchrony and improvements in lung compliance [4]. The results of three recent meta-analyses have all demonstrated that NMBA administration in ARDS patients is associated with reduced barotrauma and improved oxygenation; however, the impact on mortality remains unclear [30–32]. Thus, the NMBA use in ARDS must be individualized and may be utilized as a part of an institutional-based protocol.

### Additional applications

The neuromuscular blockade has been used in patients with status asthmaticus. However, this specific application’s use has decreased over concerns of severe weakness and critical care myopathy [33–35]. Indeed, the current SCCM clinical practice guidelines [4] suggest against the routine administration of an NMBA to patients with status asthmaticus (*weak recommendation with very low quality of evidence*). Interestingly, more recent investigations have suggested that replacing neuromuscular blockade with continuous deep sedation regimens did not change the incidence of muscle weakness in this group of patients, suggesting that prolonged immobilization and inactivity are key clinical contributors to this complication rather than solely due to the administration of NMBAs [34].

In patients with an acute brain injury, a mass occupying lesion or subsequent intracranial edema, increases in cerebral perfusion can cause a deleterious increase in intracranial pressure (ICP). However, the current SCCM clinical practice guidelines [4] could not recommend whether NMBAs were beneficial or harmful when used in patients with acute brain injury and raised ICP (*insufficient evidence*). Neuromuscular blockade may be useful in the short-term without negatively impacting hemodynamic parameters such as ICP, cerebral perfusion pressure (CPP), and blood pressure [36]. Furthermore, the avoidance of coughing, straining, and ventilator dyssynchrony during periods of the neuromuscular blockade can avoid significant increases in ICP and worsening of cerebral edema [36, 37]. The benefits of NMBAs are limited to endpoints such as reducing oxygen consumption as well as carbon dioxide production, although this practice has not been shown to improve overall outcomes and may increase the ICU length of stay, risk of pneumonia, and overall costs [37].

As in ARDS, the early use of NMBAs in sepsis may reduce in-hospital mortality [38, 39]. Current guidelines from the Surviving Sepsis Campaign [40] list the administration of NMBAs as a weak recommendation and suggest that their use may have some benefits if used within 48 h in those adult patients with sepsis-induced ARDS.

In patients who suffer an out of hospital cardiac arrest, the use of therapeutic hypothermia plays an important role in survival to discharge [41]. However, the current SCCM clinical practice guidelines [4] make no recommendation on the routine use of NMBAs for such patients (*insufficient evidence*). A complication from hypothermia is shivering, which leads to the deleterious consequences of increased metabolic rate and ICP, heat production, inflammation, and decreased brain tissue oxygen levels [42]. The American Heart Association guidelines recommend short-acting NMBAs in conjunction with appropriate use of analgesia and sedation to alleviate shivering in this setting [42, 43]. Indeed, the SCCM guidelines also suggest that NMBAs be used to manage overt shivering during therapeutic hypothermia (*weak recommendation, very low quality of evidence*).

The only neuromuscular blockade patient management recommendation that was rated as “strong” by the SCCM panel of experts was the use of lubricating drops or gel along with eyelid closure for patients receiving continuous infusions of NMBAs [4]. Additionally, targeting glucose levels less than 180 mg/dL (10 mM) and the implementation of a physiotherapy regimen during periods of neuromuscular blockade also represent “weak” recommendations. The SCCM recommendations are not mandates, and the authors clearly state that therapy should be guided by the patient’s condition, clinician experience, and equipment available in the ICU [4]. Clinical care providers must maintain an understanding of clinical pharmacology in order to weigh the clinical benefits versus the associated risks when deciding when NMBAs may suit the needs of their specific patient.

## Specific neuromuscular blocking agents

NMBAs cause skeletal muscle relaxation by blocking the transmission of impulses at the neuromuscular junction (NMJ) [44]. These agents are classified by their mechanism of action and chemical structure. Based on their methods for establishing neuromuscular blockade, there are two types: depolarizing and non-depolarizing NMBAs. The group of non-depolarizing NMBAs is further subdivided according to their structure into benzylisoquinolinium (curare, atracurium, cisatracurium, mivacurium) and aminosteroidal compounds (rocuronium, vecuronium, pancuronium). Selecting a specific NMBA in the critically ill patient depends on the indication, patient’s comorbidities (liver or renal failure), and interactions with other drugs that may enhance or prolong their action, as well as physiological changes and risk factors that may affect the pharmacokinetics of NMBAs such as age-related changes [44], hypothermia [45–47], sepsis [48–50], and metabolic or electrolyte disturbances (Table 4) [51].

**Table 4:** Neuromuscular blocking agents (adapted from Sturgess, Anaesthesia 2017 [25].)

Agent	ED_**95**_^a^ (mg/kg)	Onset time	Infusion dose (μg/kg/min)	Clinical duration	Notes
Succinylcholine	0.5–0.6	30–60 s	NR	Dose dependent; 3 × ED_95_ lasts 12–15 min	Transiently increases serum K levels by 0.5 mEq, can be used for RSII, metabolized by butyrylcholinesterase^c^
Rocuronium	0.3^b^	1.5–3 min	5–12	20–70 min	Can be used for RSII, eliminated by the liver (90%) and kidneys (10%)
Vecuronium	0.05	3–4 min	1–2	25–50 min	Active metabolites, associated with ICUAW
Mivacurium	0.08	3–4 min	5–8	15–20	Metabolized by butyrylcholinesterase^c^, associated with histamine release
Cisatracurium	0.05	4–7 min	1–3	35–50 min	Hofmann elimination
Atracurium	0.25	3–5 min	10–20	30–45 min	Metabolized by plasma esterase and Hofmann elimination, associated with histamine release
Pancuronium	0.07	2–4 min	20–40 (not recommended)	60–120 min	Active metabolites, associated with ICUAW, vagolytic effect causes tachycardia

*ED*_*95*_ effective dose that decreases the twitch by 95% from baseline, *ICUAW* intensive care unit-acquired weakness, *NR* not recommended, *RSII* rapid sequence induction and intubation

^a^Intubating dose is 2 × ED_95_

^b^1.2 mg/kg (4 × ED_95_) can be used for rapid sequence induction and intubation

^c^Also referred to as plasma cholinesterase or pseudocholinesterase

### Benzylisoquinolinium agents

Atracurium is an intermediate-acting NMBA that is metabolized through nonspecific plasma esterase-mediated hydrolysis as well as Hofmann elimination reaction in which the compound is degraded based on body pH and temperature [52]. This breakdown is nonenzymatic and occurs independent of hepatic and renal function, making this agent an attractive option in the intensive care unit in patients with renal and/or hepatic dysfunction. The Hofmann elimination reaction produces laudanosine, a compound that has been shown to cause seizure-like activity in high doses but only in animal models [53]; in fact, this complication has never been reported in humans at clinically relevant doses [54]. Intubating doses of atracurium (0.5 mg/kg or 2 × ED_95_) can cause clinically relevant histamine release, producing tachycardia, hypotension, and skin flushing [55].

Cisatracurium is the cis-cis isomer of atracurium, a feature that increases its potency four-fold, without the associated histamine release; therefore, a smaller dose is required for tracheal intubation (0.1 mg/kg or 2 × ED_95_). This intermediate-acting agent is also metabolized through organ-independent mechanisms via the Hofmann elimination reaction, making this benzylisoquinolinium drug one of the most commonly utilized NMBAs in critically ill patients who require neuromuscular blockade [54, 56, 57]. Sottile and colleagues performed a large observational study in patients with ARDS and found that when compared with vecuronium, cisatracurium was associated with increased ventilator-free days and overall ICU days but was not associated with a difference in mortality [58], suggesting cisatracurium is the preferred neuromuscular blocking agent for patients at risk for, or with, ARDS.

Unlike cisatracurium and atracurium, mivacurium is a short-acting nondepolarizing NMBA. Mivacurium was developed in the 1990s and has recently been reintroduced to the US market [59]. Antagonism of mivacurium-induced neuromuscular blockade with anticholinesterase inhibitors can shorten the duration of blockade, although paradoxical prolongation of blockade has been reported, necessitating the need for confirmation of recovery using objective monitoring [60]. Spontaneous recovery from mivacurium occurs via butyrylcholinesterase degradation within 12–20 min after administration of an intubating dose (0.25 mg/kg or 3 × ED_95_); patients deficient in this enzyme can have prolonged effects [59].

### Aminosteroidal agents

Rocuronium is an intermediate-acting NMBA and is the only nondepolarizing drug that is currently utilized in a rapid sequence induction and intubation. A dose of 1.2 mg/kg (4 × ED_95_) produces a similar average onset time to that of succinylcholine, although individual patient responses can vary [14]. Rocuronium administration is not associated with histamine release, and it has a little impact on hemodynamics. It is predominantly cleared through the biliary route, although a small portion is renally excreted and clearance can be slowed in patients with severe renal impairment [61]. Metabolism of rocuronium produces an active metabolite, 17-desacetyl-rocuronium, which has 5% of the neuromuscular blocking potency of the parent compound [62]. Allergic reactions may be a concern with the use of rocuronium as the frequency of such events is higher than with other nondepolarizing NMBA and similar to that of succinylcholine [63].

Vecuronium, like rocuronium, is an intermediate-acting NMBA with a very stable hemodynamic profile. Unlike rocuronium, higher doses do not result in significantly shorter time to onset, precluding the use of vecuronium in a rapid sequence induction and intubation. Patients with hepatic or renal impairment can experience prolonged effects from vecuronium. Furthermore, vecuronium is metabolized to 3-desacetyl-vecuronium, a compound with significant neuromuscular blocking activity [64]. Although vecuronium is not associated with hemodynamic perturbations, its active metabolites and association with ICU-acquired weakness warrant caution in the critical care setting.

Pancuronium is a long-acting aminosteroidal NMBA that can have prolonged effects in patients with organ dysfunction [61, 65]. This agent causes direct sympathomimetic stimulation and antagonizes cardiac muscarinic receptors [66], often resulting in tachycardia. Pancuronium is metabolized to three metabolites, with 3-OH pancuronium being the most clinically relevant: it has 50% of the neuromuscular blocking potency of the parent compound [67], contributing to the accumulation and prolonged duration of action with repeated pancuronium administration. Therefore, the use of pancuronium in the critical care setting is discouraged.

### Depolarizing agents

As the only depolarizing NMBA available, succinylcholine produces neuromuscular blockade by competing with acetylcholine (ACh) at the postsynaptic nicotinic receptors. Following the administration, succinylcholine produces a reliably rapid blockade and can be used to facilitate rapid sequence induction and tracheal intubation. Its use is associated with skeletal muscle fasciculations after administration, and waiting at least 30 s after the cessation of fasciculations should provide optimal blockade for endotracheal intubation [68, 69]. Succinylcholine is a known trigger for malignant hyperthermia and causes a transient increase in plasma potassium levels by 0.5–1.0 mEq/L [70, 71]. This hyperkalemic response can be exaggerated in patients with upregulated extrajunctional nicotinic acetylcholine receptors (nAChRs). The proliferation of such receptors occurs in patients with prolonged immobility, acute burns, stroke with paralysis, spinal cord injury, demyelinating disorders, and even sepsis [72]. This feature is of particular concern in the critically ill patient as the duration of ICU stay has been correlated with the risk of hyperkalemia (potassium ≥ 6.5 mEq/L) [73]. Therefore, clinicians must be aware of recent serum potassium concentration and relevant patient history regarding neuromuscular pathology prior to administration of succinylcholine in the ICU.

## Reversal agents (pharmacologic antagonists)

In the perioperative setting, pharmacologic antagonism of neuromuscular blockade is routinely used to restore baseline function and reduce the risk of postoperative residual paralysis [74]. Current trends in ICU management most often allow for spontaneous recovery, and pharmacologic reversal is uncommon. Nonetheless, intensivists should be familiar with the antagonists for this potentially harmful class of medications in order to restore neuromuscular function in patients.

### Acetylcholinesterase inhibitors

Neostigmine and edrophonium antagonize the action of NMBAs by preventing the action of the enzyme acetylcholinesterase. This enzyme breaks down ACh in the neuromuscular junction, and its inhibition results in the accumulation of ACh that competes with NMBA for binding sites on postsynaptic receptors. Neostigmine should not be utilized to reverse moderate levels of neuromuscular blockade (train-of-four count < 1–3) but should be reserved for situations with the train-of-four count > 3 (Table 5). Median recovery time is approximately 15 min, although significant variability exists among patients and clinical scenarios [75]. Because the increase in ACh also affects muscarinic receptors, an antimuscarinic drug such as glycopyrrolate is typically co-administered to avoid side effects such as significant bradycardia and bronchoconstriction [76].

**Table 5:** Levels of neuromuscular block

Level of block	Depth of block	Objective measurement at APM	Subjective evaluation with PNS at APM
Level 5	Complete	PTC = 0	PTC = 0
Level 4	Deep	PTC ≥ 1, TOFC = 0	PTC ≥ 1, TOFC = 0
Level 3	Moderate	TOFC = 1–3	TOFC = 1–3
Level 2b	Shallow	TOFR < 0.4	TOFC = 4, TOF fade present
Level 2^a^	Minimal	TOFR = 0.4–0.9	TOFC = 4, TOF fade undetectable
Level 1	Adequate recovery	TOFR ≥ 0.9	Cannot be determined

*APM* adductor pollicis muscle, *NMB* neuromuscular blockade, *PNS* peripheral nerve stimulator, *PTC* posttetanic count, *TOF* train of four, *TOFC* train-of-four count, *TOFR* train-of-four ratio

^a^Subjective evaluation of the depth of neuromuscular block is not recommended, but it is included as an interim transition from current practice to the preferred, objective monitoring-based practice. Reproduced with permission [95]

### Sugammadex

Rocuronium and vecuronium can be antagonized with sugammadex, a gamma-cyclodextrin compound that encapsulates and binds these NMBAs. This encapsulation process occurs in the plasma, creating a concentration gradient that facilitates the transfer of aminosteroidal NMBA from the neuromuscular junction back into the circulation. The tightly bound, inactive sugammadex-aminosteroidal complex is then excreted in the urine [77]. Sugammadex has the unique ability to reverse deep or profound levels of neuromuscular blockade and restore neuromuscular function faster than spontaneous recovery from succinylcholine [78], although this rescue technique should not supplant prudent airway management [16]. It is not approved for use in patients with a creatinine clearance < 30 ml.min^-1^; however, several studies have reported its use in patients with a significant renal disease without complications [79–81]. In addition, the NMBA-sugammadex complex can be removed via standard dialysis techniques [82]. Concern exists over hypersensitivity reactions following sugammadex administration [83]; however, the overall incidence of such events remains low and rarely impacts routine clinical care [84]. While not currently widely used in the critical care setting, its use may expand as new evidence emerges describing its use as a rescue therapy for residual blockade [85] and its role in reducing the incidence of reintubation [86] and promoting enhanced recovery protocols in the ICU [87]. In an effort to reduce the incidence of residual weakness and recurrence of neuromuscular blockade, we recommend dosing sugammadex based on actual body weight (rather than ideal body weight) and utilizing neuromuscular monitoring to confirm adequate recovery prior to extubating the patient’s trachea.

## Determining the level of neuromuscular blockade

### Subjective evaluation with a peripheral nerve stimulator

Titrating appropriate levels of neuromuscular blockade may be essential to avoid prolonged paralysis in the ICU [88]. While the use of continuous NMBA infusions rather than intermittent boluses was reported to minimize the risk of prolonged paralysis [89], current guidelines also suggest that the use of a peripheral nerve stimulator (PNS) can be a useful tool, when combined with other clinical assessment, to determine adequate neuromuscular blockade. Indeed, a PNS is utilized by a majority of institutions to guide neuromuscular blockade in the critical care setting [90]. While expert opinion has driven such implementation [91, 92], a large randomized, prospective study demonstrated that utilizing a PNS reduced the incidence of prolonged muscle recovery and the overall amount of NMBA administered [93]. Furthermore, the use of a PNS has been shown to achieve overall cost savings, primarily through less drug being needed to maintain the desired level of paralysis [94]. An international panel of experts recently recommended at least the use of a PNS whenever neuromuscular blockade is utilized, although quantitative monitors are the only means of reliably confirming recovery [95].

**Fig. 1:**
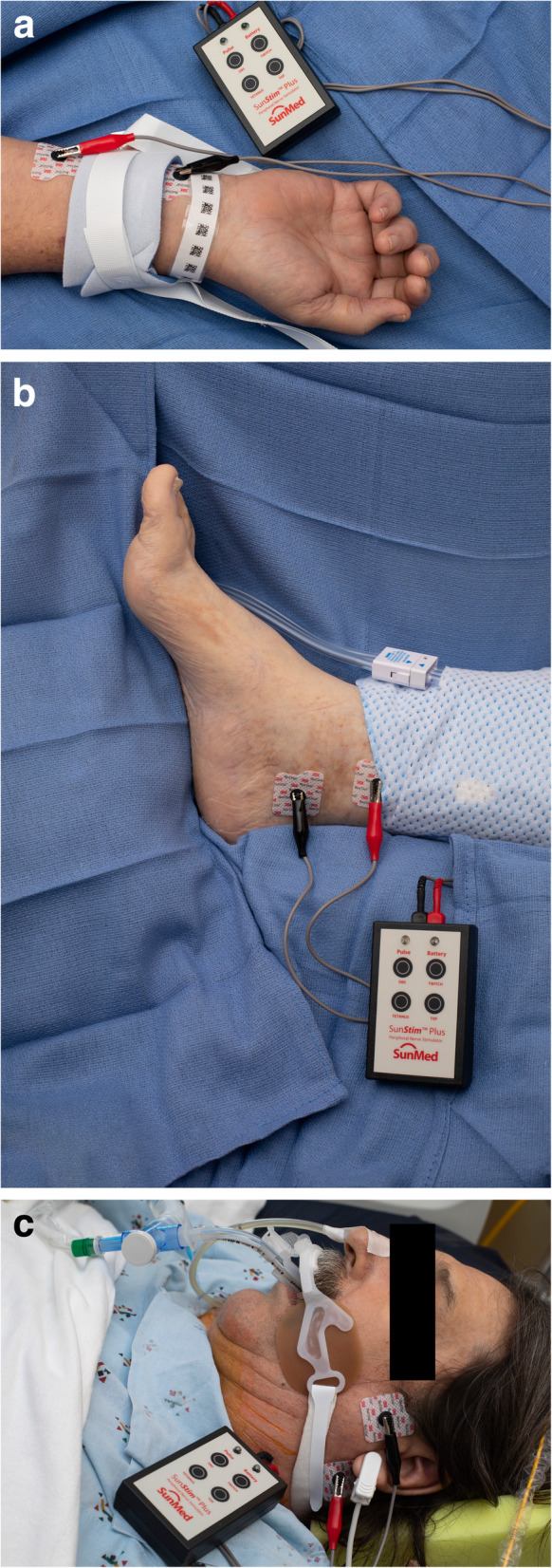
**a** Peripheral nerve stimulator over the ulnar nerve of a patient with limb restraints. **b** Peripheral nerve stimulator over the posterior tibial nerve. **c** Peripheral nerve stimulator over the facial nerve

Several obstacles and limitations exist when utilizing a PNS. Significant inter-observer variability can exist when using a PNS as the providers may visually or tactilely evaluate the response to train-of-four stimulation [96]. Different muscle groups will have different sensitivity to NMBA administration, leaving the site of monitoring particularly important when determining the level of blockade (Fig. 1) [96]. The detection of fade, a feature that signifies some degree of the residual blockade and incomplete restoration of baseline function, is challenging even for the experienced anesthesiologist who evaluates multiple train-of-four stimulations daily [97]. Such challenges are magnified in the ICU setting, as providers may have little or infrequent experience with using a PNS. Additionally, patients with significant perspiration and tissue edema in the ICU can present obstacles to performing adequate neurostimulation.

### Quantitative monitors

While not common practice, handheld quantitative (objective) monitoring technology is expanding and improving. The use of these devices is increasing in the perioperative arena, and their application to guide administration of NMBAs and confirm recovery from neuromuscular blockade perioperatively has recently been recommended by a panel of experts [95]. Quantitative monitoring carries a distinct advantage over the use of a PNS in that it objectively measures and calculates the train-of-four count and ratio, rather than relying on visual or tactile assessment by clinicians. Transitioning from subjective evaluation to precisely measuring the level of blockade with quantitative monitoring represents a significant improvement in neuromuscular blockade management in the critical care setting and reduces inter-observer variability. Additionally, quantitative monitors are the only reliable means to confirm adequate recovery from neuromuscular blockade prior to tracheal extubation, a clinical prerequisite that is vital in the vulnerable ICU patient population. Regardless of whether reversal agents are utilized or if clinicians rely on the NMBAs’ pharmacokinetics to recover spontaneously, adequate recovery must be documented to avoid complications of residual paralysis such as oropharyngeal dysfunction and critical respiratory events [98, 99].

**Fig. 2:**
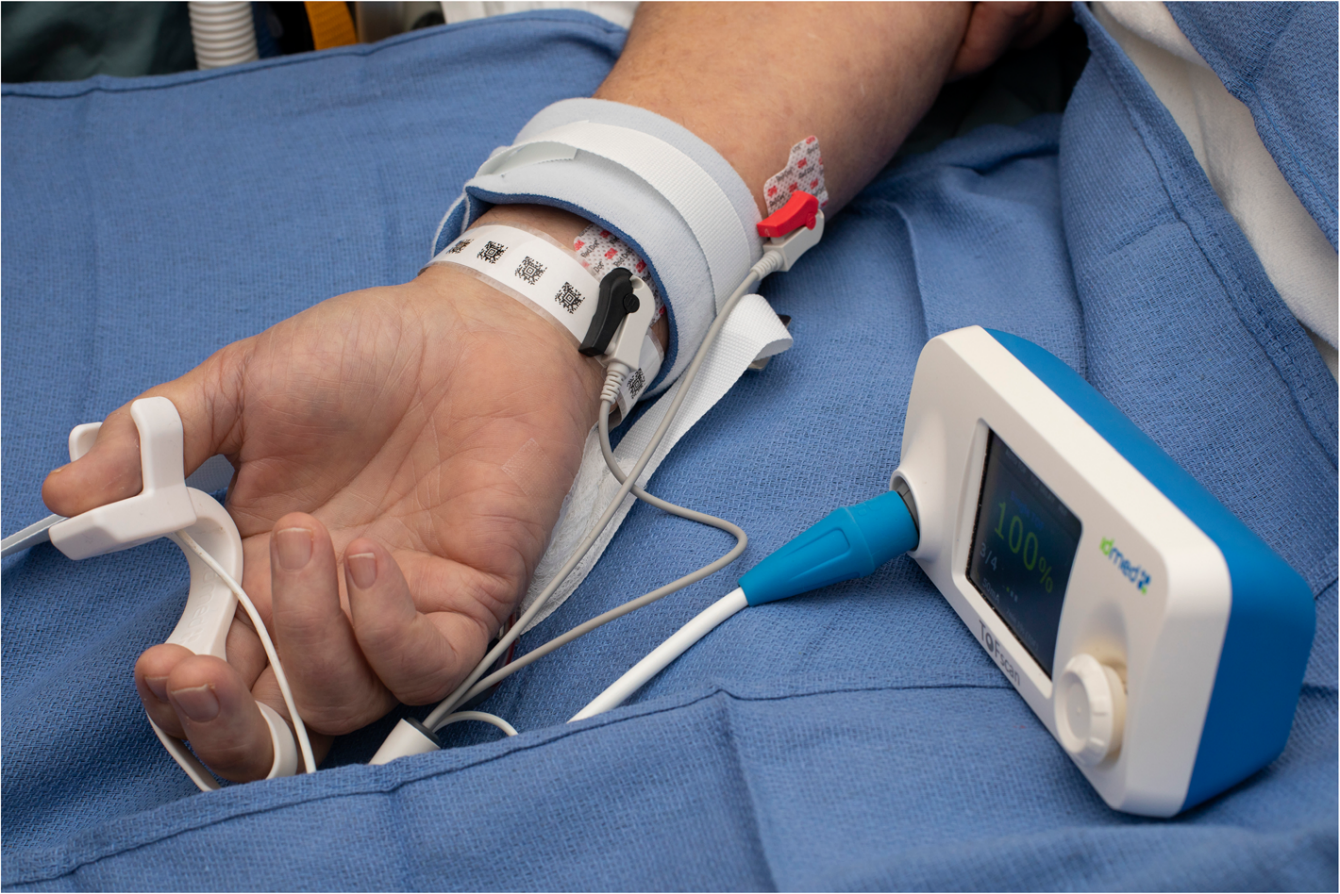
The acceleromyography-based TOFscan device (Drager Technologies, Canada) measuring the response to neurostimulation of the adductor pollicis muscle

**Fig. 3:**
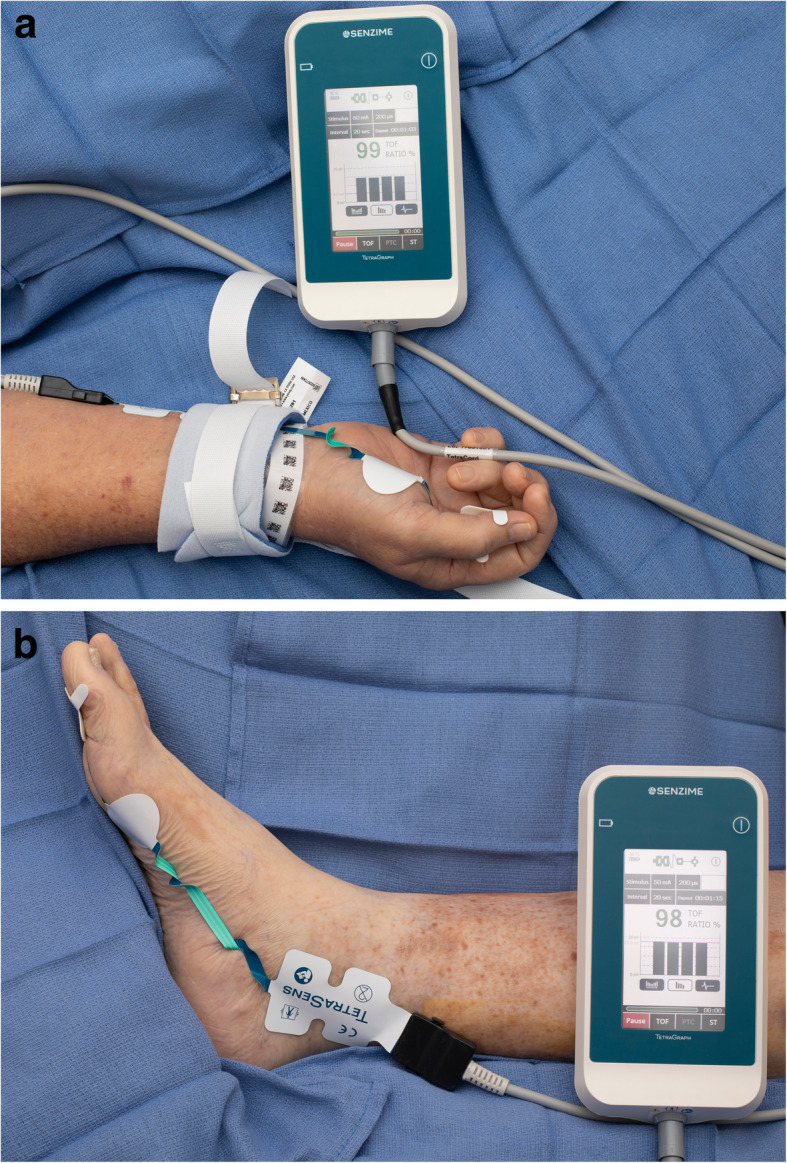
**a** The electromyography-based TetraGraph device (Senzime AB, Uppsala, Sweden) measuring the response to neurostimulation of the adductor pollicis muscle. **b** The electromyography-based TetraGraph device (Senzime AB, Uppsala, Sweden) measuring the response to neurostimulation of the flexor hallucis brevis muscle

**Fig. 4:**
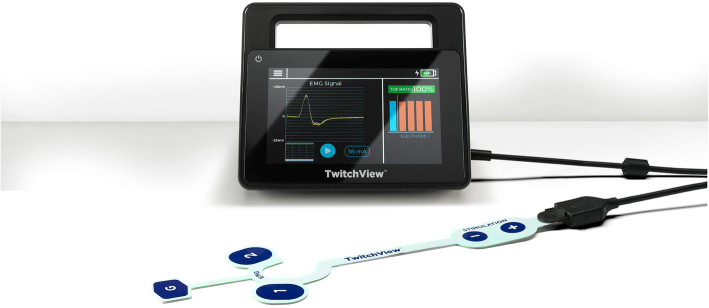
The electromyography-based TwitchView device (Blink Device Company, Seattle, WA)

Quantitative monitors can be categorized based on the mechanism by which the train-of-four count and/or ratio are measured [100]. Acceleromyography (AMG) is the most commonly utilized quantitative monitor and relies on Newton’s second law that states force is proportional to acceleration. By measuring the acceleration of the monitored muscle group, AMG devices can calculate the train-of-four ratio and confirm adequate recovery from neuromuscular blockade. Kinemyography (KMG) measures the degree of bending of a sensor strip positioned between the thumb and index finger after neurostimulation. Both KMG and AMG require the muscle group being monitored to move freely without restriction as they utilize integrated piezoelectric motion sensors to quantify the response to neurostimulation. Electromyography (EMG) does not require freely moving muscle groups, as it measures the electrical response of the muscle upon neurostimulation. This response is proportional to the force of contraction, without requiring an actual contraction. Because of this characteristic, EMG may be suitable for confirming recovery for the neuromuscular blockade in the critical care setting that commonly utilizes limb restraints (and in clinical settings in which the use of AMG- or KMG-based monitors is limited). Similar to using a PNS, EMG- and AMG-based quantitative monitors can also be utilized to monitor other muscle groups (facial, foot) if the hand is unavailable (Figs. 2, 3, and 4).

## Sedation strategies

A comprehensive review of sedation strategies in the ICU is beyond the scope of this review. Nonetheless, vigilance is warranted in maintaining adequate sedation when NMBAs are utilized in order to avoid unintended patient awareness and recall. Clinicians must recognize markers of inadequate sedation such as tachycardia, hypertension, diaphoresis, and ventilator dyssynchrony. While the use of processed electroencephalography (EEG) has been shown to decrease the risk of intraoperative awareness in high-risk surgical patients [101], current guidelines make no recommendations regarding the use of such technology in the critical care setting when NMBAs are administered [4]. However, we recognize that the utilization of processed EEG monitors at the bedside of ICU patients receiving NMBA infusions is becoming more common.

## Complications from neuromuscular blockade

The use of NMBAs in the ICU setting risks numerous complications. Most notably, neuromuscular blockade results in prolonged patient immobility that can lead to the development of acquired weakness, myopathy, pressure ulcers, nerve injuries, and risk of deep venous thrombosis (DVT) [42]. Because the critically ill patient has an increased risk of DVT in their lower extremities compared with other hospitalized patients, special attention should be given to this potentially preventable complication [102, 103]. Boddi et al. found in their multivariate analysis that NMBAs were the strongest independent predictor for DVT incidence in the ICU [102]. Special care and consideration should be given to patients who receive NMBAs with regard to optimizing DVT prevention.

Multiple studies have shown that there is a correlation between ICU-acquired weakness (ICUAW) and neuromuscular blockade [34, 104, 105]; however, there is a lack of well-designed clinical trials confirming this relationship [106]. ICUAW represents a heterogeneous term that has been used to describe varying conditions such as critical illness polyneuropathy (CIP), critical illness myopathy (CIM), and critical illness neuromyopathy (CINM), a diagnosis that is based on electrophysiologic testing. The etiology of such states is often multifactorial, and the reported outcomes are also heterogeneous. A recent meta-analysis suggested a modest association between NMBA use and ICUAW [107]; however, the studies that were included with a strong association have a high risk of bias, and the studies with the lowest risk of bias that performed multivariable adjustment suggested a small, but not significant association. Nevertheless, the authors’ sensitivity analysis showed an increased risk of CIP in septic shock patients exposed to NMBAs, and consistent with previous studies [108, 109], the association may be proportional to the severity of the sepsis; therefore, the authors recommended to be cautious and target early use. Association between the ICUAW and NMBA use remains controversial. Well-designed trials should be performed to determine if the use of NMBAs is an independent cause of ICUAW.

Unintended (or accidental) awareness and recall are also a major concern during the use of NMBAs [110, 111]. In patient interviews, feelings of dying, being tied down, and fear were expressed with the concomitant use of NMBAs. Though the exact regimen of sedation and analgesia was not known in these patients, this complication reinforces the importance of providing proper sedation and not only relying on a single monitor, such as processed electroencephalography (pEEG). Rather, clinicians must assimilate multiple markers of sedation such as unexplained tachycardia and hypertension, ventilator dyssynchrony, and tearing to avoid this complication.

Once patients’ tracheas are extubated, the most feared complication is hypoxemia and the subsequent need for reintubation. NMBAs have been known to cause adverse pulmonary outcomes [112] such as decreased inspiratory flow [113], residual paralysis [114], and impaired airway protective reflexes [99]. Such clinical features place patients at increased risk of upper airway obstruction, pneumonia, and reintubation. Identification of patients who may be at risk for adverse respiratory events was highlighted by Stewart and colleagues in 2016 [115]. These investigators found that > 30% of patients in the post-anesthesia care unit had residual neuromuscular blockade, and this risk was increased with older age, abdominal surgery, and surgery duration greater than 90 min [115]. Patients with obstructive sleep apnea (OSA) who receive NMBAs may also be at higher risk for postoperative respiratory complications compared to patients who do not have OSA [116]. While this risk stratification has not been applied to the ICU setting, such clinical predictors may prove useful and applicable in critically ill patients. Additionally, the use of a “leak test” has been proposed to identify patients at risk for post-extubation stridor that can result from laryngeal edema [117]. While the incidence of this complication has been found to be as high as 22% [118], a recent prospective, multicenter trial found it to be less than 10% [119]. Interestingly, these authors propose that the increasing use of neuromuscular blockers at the time of endotracheal intubation may be a contributing factor to this decline [119]. Regardless, vigilance is warranted following extubation as post-extubation stridor is a significant predictor of prolonged mechanical ventilation and prolonged ICU length of stay [120, 121].

## Conclusions

While the administration of NMBAs can prove to be a life-saving therapy in select critically ill patients, these medications have unique inherent risks as well. However, by understanding the pharmacology, dosing, drug interactions, side effects, and monitoring techniques, clinicians can safely maximize the benefits. As there are few prospective studies that support improved long-term outcomes for patients in the ICU, the administration of NMBAs should be limited to facilitating endotracheal intubation, prevention of shivering following therapeutic hypothermia, and avoiding increases in intracranial pressure in patients at risk associated with coughing or ventilator dysynchrony. Moreover, residual weakness following the use of NMBAs in the ICU is a particular concern, given this vulnerable population. This complication may occur more frequently in the ICU, given the abundance of patients with significant organ dysfunction and delayed drug (NMBA) elimination. We recommend continuous vigilance when NMBAs are used in critically ill patients, selecting the most appropriate NMBA for each individual clinical scenario, evidence-based protocols that ensure adequate sedation and analgesia, appropriate equipment for assessing the degree of neuromuscular blockade, and aggressive physical therapy regimens during periods of reduced mobility. Such a multifaceted approach can improve patient safety when NMBAs are utilized in the ICU and reduce associated complications.

## Data Availability

Not applicable

## Abbreviations

ACh: Acetylcholine
AMG: Acceleromyography
ARDS: Acute respiratory distress syndrome
ASA: American Society of Anesthesiologists
CIM: Critical illness myopathy
CINM: Critical illness neuromyopathy
CIP: Critical illness polyneuropathy
DVT: Deep venous thrombosis
ED95: Effective dose that decreases the twitch by 95% from baseline
EEG: Electroencephalography
EMG: Electromyography
GRADE: Grading of Recommendations Assessment, Development, and Evaluation
ICP: Intracranial pressure
ICU: Intensive care unit
ICUAW: Intensive care unit-acquired weakness
KMG: Kinemyography
nAChR: Nicotinic acetylcholine receptors
NAP4: 4th National Audit Project
NMBA: Neuromuscular blocking agent
NMJ: Neuromuscular junction
OR: Operating room
OSA: Obstructive sleep apnea
PaO_2_/FiO_2_: Partial pressure of oxygen to fraction of inspired oxygen
pEEG: Processed electroencephalography
PNS: Peripheral nerve stimulator
RSII: Rapid sequence induction and intubation
SCCM: Society of Critical Care Medicine
